# Liver Abscess Due to Dropped Appendicolith after Laparoscopic Appendectomy

**DOI:** 10.5334/jbr-btr.935

**Published:** 2015-12-30

**Authors:** K. Muyldermans, C. Brussaard, I. Willekens, J. de Mey

**Affiliations:** 1 Department of Radiology, University Hospital Brussels, Laarbeeklaan 101, 1090 Brussels, Belgium

**Keywords:** appendicolith, Liver abscess

## Abstract

The lifetime risk of appendicitis is 6 to 7 % [1]. When appendicitis is clinically suspected, an appendicolith can be found in 30% of the patients [2]. An appendicolith may be retained post-operatively (‘dropped appendicolith’) due to previous perforation, non-recognition during surgery or the impossibility to remove it. Abscesses that result from ectopic appendicoliths tend to occur paraceacally in the vicinity of Morrison’s pouch and should be removed to prevent abscess development and possible overt sepsis [3]. As far as we know, we describe the first documented case of an intrahepatic localization of a dropped appendicolith causing a liver abscess.

## Introduction

The lifetime risk of appendicitis is 6 to 7 % [1]. When appendicitis is clinically suspected, an appendicolith can be found in 30% of the patients [2]. An appendicolith may be retained post-operatively (‘dropped appendicolith’) due to previous perforation, non-recognition during surgery or the impossibility to remove it. Abscesses that result from ectopic appendicoliths tend to occur paraceacally in the vicinity of Morrison’s pouch and should be removed to prevent abscess development and possible overt sepsis [3]. As far as we know, we describe the first documented case of an intrahepatic localization of a dropped appendicolith causing a liver abscess.

## Case report

**Figure 1A,B:**
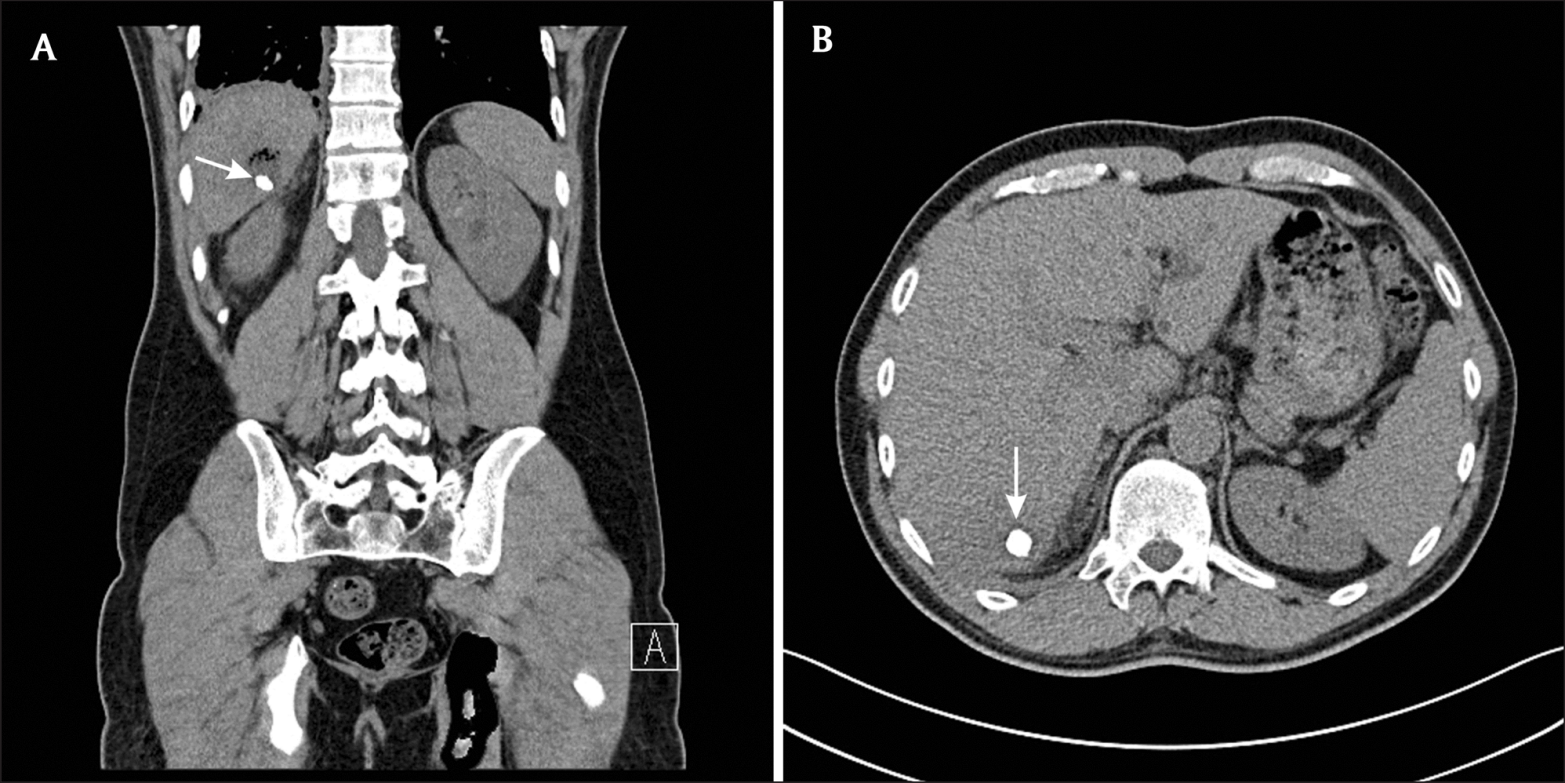
Coronal and axial reconstruction of the non-contrast enhanced CT-scan performed at readmission. Intrahepatic localization of the former seen appendicolith surrounded by a hypodense zone with air bubbles, indicating pus.

**Figure 2:**
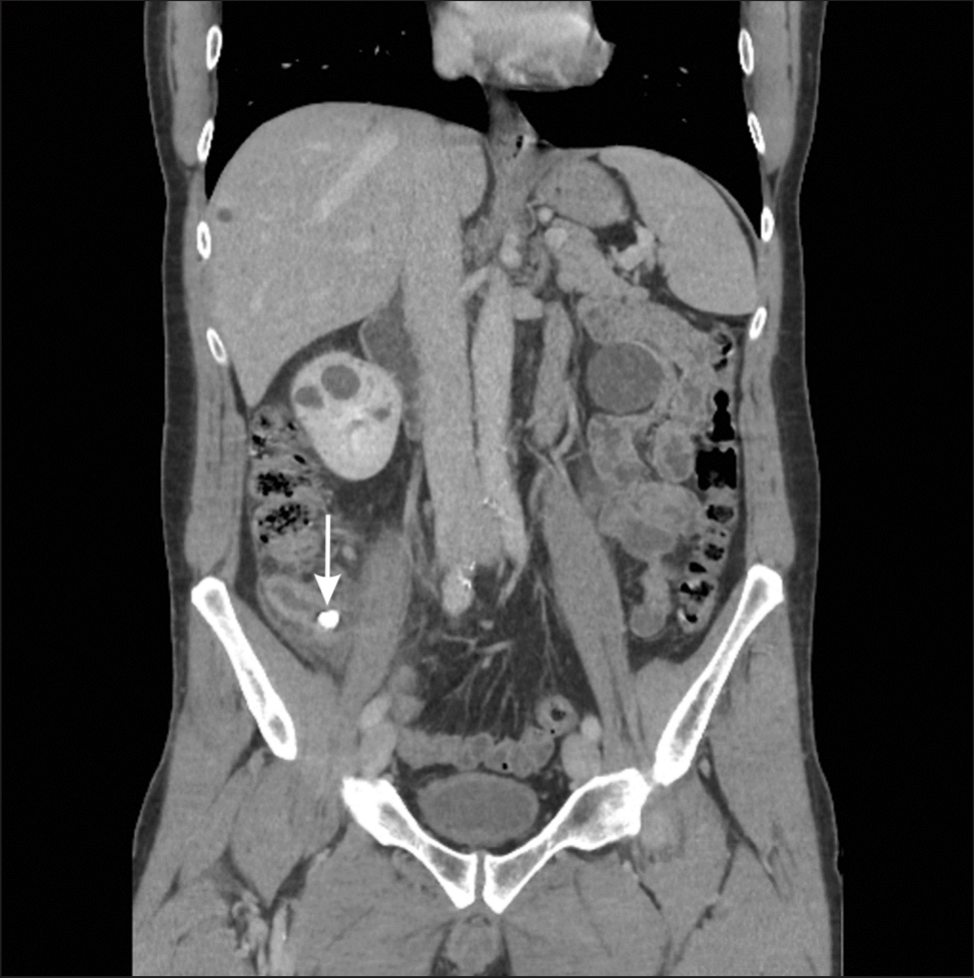
Coronal reconstruction of the initial contrast-enhanced CT performed at the acute onset of appendicitis. The appendix shows a thickened wall, fat stranding, free fluid and the embedded appendicolith. No obvious signs of a macroscopic interruption of the appendiceal wall was noted.

A 46-year old man presented to the emergency department with pain localized to the right costovertebral angle and associated shoulder pain. Laboratory findings showed raised inflammatory parameters (C-reactive Protein (CRP) 116 mg/dL, normal range 0–1.2 mg/dL). The patient had no fever (36°C). A non-contrast-enhanced CT-scan was performed to exclude kidney stones. No urinary tract calculi could be revealed. However, in liver segment 7, a high-density structure was retained surrounded by a hypodense zone of 25 mm, containing small air bubbles suggestive for an intrahepatic abscess (Figure 1A and 1B). Review of the contrast enhanced CT-scan performed two weeks earlier on the occasion of an acute appendicitis learned that this intrahepatic calcification had the same characteristics (800 Hounsfield Units, 10 mm, round shape) as the appendicolith on the previous scan (Figure 2). At that time, the patient was treated with laparoscopy, which revealed a necrotizing appendicitis with a small covered perforation. Following a five day course of antimicrobial therapy the patient was discharged home.

Due to the recent laparoscopic appendectomy for acute appendicitis, the CT-findings of this admission suggest a dropped appendicolith, which had spontaneously migrated into the liver parenchyma causing an intrahepatic abscess. There are no arguments for an iatrogenic lesion of the liver capsule during the recent appendectomy. During the second laparoscopic exploration, the appendicolith was extracted and the abscess was drained. Microbiology was positive for Escherichia coli. Intravenous antibiotics were administered over the following four days and the patient discharged. Up to now, the patient has remained well.

## Discussion

An appendicolith consists of thickened faecal material, mucus with embedded calcium phosphate and inorganic salts [4]. It can cause an obstruction of the lumen of the appendix. Its local mass effect may damage the mucosa by compromising the vascular circulation and can cause abnormal bacterial proliferation distal from the appendicolith, resulting in an inflammatory reaction and possible raise in intraluminal pressure. Moreover, if ischemia of the appendiceal wall occurs, this can lead to a perforation. When an appendicolith is present, it is often associated with more severe appendicitis and a higher perforation rate [5].

CT is useful for identifying patients with complicated appendicitis. Fat stranding and free fluid on CT are significant for complicated appendicitis and CT is a powerful tool for identifying patients with complicated appendicitis preoperatively [6].

On CT-scan, a dropped appendicolith presents mostly as a zone of high attenuation of less than 1 cm in diameter. The dropped appendicolith can be associated with an abscess mostly close to the caecum, Morrison’s pouch or in the pelvis [3].

As described in a case report by Whalley et al. CT images showed a right subphrenic collection, which was indenting the right lobe of the liver, with an appendicolith in the middle [7].

A case report by Michelle et al. described the typical sonographic findings of a perihepatic abscess caused by dropped appendicoliths. They revealed that CT appearance could mimic an intrahepatic lesion, although appendicoliths contained in the abscess would be an atypical finding for an intrahepatic abscess. They showed the benefit of ultrasound to differentiate between an intrahepatic and perihepatic location, the latter which is more frequent [8]. Although, in our case, CT scan showed that the appendicolith actually migrated spontaneously into the liver parenchyma and served as a nidus for intrahepatic abscess formation. Ultrasound was not performed in our institution.

A dropped appendicolith with abscess must be treated by open or laparoscopic surgery removing the appendicolith and draining of the abscess. Successful percutaneous removal of a dropped appendicolith and concomitant abscess drainage has been described in literature. In the setting of abscess drainage with retained appendicolith, abscess recurrence is inevitable [9]. At surgery for acute appendicitis with an incorporated appendicolith, a dropped appendicolith could be avoided by double ligature [10]. However, this method gives no protection against a fallen appendicolith in case of a macroscopic interruption of the appendical wall, as in the presented case.

## Conclusion

Relapsing inflammatory parameters after appendectomy can be caused by a dropped appendicolith. Carefull reporting by radiologist and surgeons on macroscopic interruption of the appendicael wall helps the radiologist to stratify the risk of a dropped appendicolith in a follow-up examination. Besides migration of an appendicolith in the free intra-abdominal space, it can spontaneously migrate into the liver parenchyma and serve as a nidus for intrahepatic abscess formation.

## Competing Interests

The authors declare that they have no competing interests.
